# Neuromuscular Correlates of the Contralateral Stretch-induced Strength Loss

**DOI:** 10.1249/MSS.0000000000002677

**Published:** 2021-04-08

**Authors:** GIUSEPPE CORATELLA, EMILIANO CÈ, CHRISTIAN DORIA, MARTA BORRELLI, STEFANO LONGO, FABIO ESPOSITO

**Affiliations:** 1 Department of Biomedical Sciences for Health (SCIBIS), Università degli Studi di Milano, Milan, ITALY; 2 IRCSS Galeazzi Orthopedic Institute, Milan, ITALY

**Keywords:** H-REFLEX, M-WAVE, SURFACE EMG, V-WAVE, PASSIVE STRETCHING, NERVE STIMULATION

## Abstract

**Purpose:**

The current study investigated the effects of unilateral passive stretching on the neuromuscular mechanisms involved in the force-generating capacity of the contralateral muscle.

**Methods:**

Twenty-six healthy men underwent unilateral passive stretching of the plantarflexors (5 × 45 s on + 15 s off; total stretching time, 225 s). Before and after the stretching protocol, contralateral ankle range of motion, maximum voluntary contraction (MVC) of the plantarflexors, and surface electromyographic root-mean-square (sEMG RMS) of the soleus and the gastrocnemii muscles were determined. Concurrently, V-wave, maximum and superimposed H-reflex, and M-wave were elicited via nerve stimulation to estimate the supraspinal, spinal, and peripheral mechanisms, respectively. sEMG RMS, V-wave, and H-reflex were normalized to the M-wave.

**Results:**

After passive stretching, contralateral ankle range of motion was increased (+8% [1%/15%], effect size [ES] = 0.43 [0.02/0.84], *P* < 0.001), MVC of the plantarflexors was decreased (−9% [−21%/−2%], ES = −0.96 [−1.53/−0.38], *P* < 0.001), and the sEMG RMS/M-wave of the soleus and the gastrocnemii muscles was decreased (≈−9%, ES ≈ −0.33, *P* < 0.05). Concurrently, the V-wave/M-wave superimposed was decreased in all muscles (≈−13%, ES = −0.81 to −0.52, *P* < 0.05). No change in H-reflex/M-wave and M-wave was observed under both maximum and superimposed condition. The decrease in the MVC and the sEMG RMS of the contralateral muscle was accompanied by a decrease in the V-wave/M-wave but not the H-reflex/M-wave ratios and the M-wave.

**Conclusions:**

The present outcomes suggest that only supraspinal mechanisms might be involved in the contralateral decrease in the maximum force-generating capacity.

Passive static stretching in sport and rehabilitation can help to improve joint range of motion (ROM), albeit it is accompanied by an acute reduction in the force-generating capacity of the stretched muscle (SM) (1,2). To explain the simultaneous increase in ROM and decrease in force-generating capacity, neuromuscular mechanisms, including alteration in the afferent feedback by type Ia, type II (muscle spindles) (3), type III (mechanoreceptors), and type IV (metabo-/nociceptors) fibers (4,5), and mechanical mechanisms, including a decrease in muscle–tendon unit stiffness (6–8), have been proposed. Specifically, the neuromuscular factors may have their origin in supraspinal inhibition and reduction in spinal reflex excitability, and/or be of peripheral origin, i.e., possible impairment in the events involved in excitation–contraction coupling processes; however, their contribution to reducing contractile force-generating capacity remains to be elucidated (9,10).

Interestingly, the effect of passive stretching may affect the homologous contralateral muscle (CM) not directly exposed to passive stretching (11). Although several studies have shown an increase in ROM in the CM (12–15), inconsistent results for the force-generating capacity have been observed with some studies reported no change (13,14,16), whereas others showed a reduction in force-generating capacity in the CM (13,17–19). Several possible mechanisms merging in a final common end, i.e., decrease in contralateral α-motoneuron pool excitability, have been proposed to explain the crossover effect in the CM: (i) a reduction in stretch–reflex sensitivity involving the muscle spindles via interneurons interposed in the crossed pathway (13,17,20), (ii) an increase in the inhibitory afferent feedback from the SM involving mechanoreceptors and nociceptors (13,17,19), and consequently (iii) an increase in stretch tolerance (19). Because the CM is not directly involved in the passive stretching maneuver, neuromuscular rather than mechanical factors have been shown to be involved (19). In addition, analyzing the force signal and concomitantly using an interpolated-twitch technique, central but not peripheral mechanisms were found responsible for the drop in the force-generating capacity in the CM, although the origin of the central neuromuscular mechanisms was not investigated (19).

To examine in depth the origin of the central neuromuscular mechanisms, the analysis of the surface electromyography (sEMG) signal coupled with peripheral nerve stimulation could be used to assess the passive stretching-induced effects on the force-generating capacity in the CM (21). More in detail, the nerve stimulation can evoke the H-reflex, M-wave, and V-wave to explore the level of the neuromuscular mechanisms (21–23). In particular, the H-reflex overall reflects the response of the motoneuron pool to a volley from large-diameter primary muscle spindle afferents (21). The M-wave represents the compound muscle action potential and is usually elicited to estimate the ability of the action potential to propagate across the sarcolemma (22). Remarkably, when evoking the M-wave with a supramaximal stimulation during maximum voluntary contraction (MVC), the wave is followed by a reflexive response, the V-wave (23,24). This response results from the motoneuron activation induced by Ia afferents after the pathway has been cleared by the collision of the antidromic and orthodromic waves (24,25). Such an evoked response is a methodological variant of the H-reflex, and its amplitude depends on the number of spinal motoneurons recruited and on their firing frequency during the MVC (25). As such, the V-wave amplitude reflects both spinal processes via reflex excitability and pre- and postsynaptic inhibition, and the level of neural drive in the descending corticospinal pathways (26). Hence, by assessing the H-reflex during MVC concomitantly with the V-wave, an index of efferent spinal motor output can be retrieved (22). The only study that partially used this approach, examining only spinal responses, found no difference in spinal excitability after a stretching bout in the CM (27).

To date, this systematic approach has never been used to investigate the effects of acute passive stretching on the mechanisms that could contribute to the loss in force-generating capacity in the CM. Therefore, the present study aimed to examine in depth the origin of the possible neuromuscular mechanisms associated with the change in force-generating capacity in the CM, by eliciting the H-reflex, M-wave, and V-wave. Because previous studies showed no change in spinal excitability (27) and M-wave (19), we hypothesized that a possible decrease in the force-generating capacity in the CM could be associated with a decrement in the V-wave.

## MATERIALS AND METHODS

### Study Design

For this cross-sectional, within-subject study, the sample size calculation was based on a previous investigation, considering the decreases in force-generating capacity of the CM as the reference parameter (18,19) and computed using statistical software (G-Power 3.1, Düsseldorf, Germany), using the *t*-test family. Cohen’s *d* effect size (ES) ~1.17 was computed using the referenced studies, a two-tail effect, *α* = 0.05, and a required power (1 − β) = 0.90; the desired sample size resulted in 10 participants. However, given the procedures we used and the possible high variability in the signals recorded, 26 participants were recruited to decrease any possible risk of bias.

### Participants

Twenty-six healthy men (age, 23 ± 4 yr; stature, 1.77 ± 0.10 m; body mass, 75 ± 11 kg; mean ± SD) volunteered for the present study. The participants were recreationally active. Inclusion criteria were no evident orthopedic and/or neurological pathologies, no lower-limb muscular or joint injuries in the previous 6 months, and no involvement in a systematic passive stretching program in the previous 6 months. The local University Ethics Committee approved the study protocol (CE 27/17); the study was performed in accordance with the principles of the latest version of the Declaration of Helsinki. The participants gave their written, informed consent after receiving an explanation of the purpose of the study and the experimental procedures. The participants were free to withdraw from the study at any time.

### Experimental Procedures

The participants were tested at the same time of the day in a climate-controlled laboratory (temperature, 20°C ± 1°C; relative humidity, 50% ± 5%) to minimize confounders due to circadian rhythms. Following previous procedures (19), the participants came to the laboratory three times. During the first session, they were familiarized with the experimental setup and the passive stretching protocol, i.e., MVC and nerve stimulation technique procedures. Skin landmarks (moles, scars, and angiomas) and the position of the angle transducer, sEMG, and stimulation electrodes were mapped on a transparency sheet for accurate electrode repositioning consistency within the same area (19). The second and the third sessions were randomized. Out of these two, one session served as a control in which the testing procedures were performed without any intervention in both limbs and replicated after a period equal to the duration of the stretching protocol. During the other visit, the participants underwent unilateral passive stretching of the plantarflexors, and the testing procedures were assessed first in the CM and then in the SM (19) because passive stretching-induced strength loss in the SM is known to last up to 2 h (28). The order of which leg was stretched first was randomized for the second session and was maintained for the third session. As such, in the control session, the SM and the CM were identified as the SM_CON_ and the CM_CON_.

The stretching protocol was performed unilaterally on the plantarflexors by the same experienced operator, and the stretched limb was randomized. Plantarflexors MVC and sEMG root-mean-square (RMS), V-wave, H-reflex, and M-wave were measured in the gastrocnemius medialis, the lateralis, and the soleus muscles of both limbs. Nerve stimulation intensity was determined to evoke H-reflex and M-wave responses at rest in all muscles. The participants performed a standardized warm-up (10 × 2-s contractions at 50% MVC determined during the familiarization session) to determine MVC and to evoke superimposed responses.

In a random order, the baseline assessments of MVC in both CM and SM were separated by at least 5 min of passive stretching. Separate nerve stimulations were used to evoke H-reflex, M-wave, and V-wave responses in the tibialis anterior only. The participants performed a standardized warm-up before MVC and to evoke superimposed responses. Two stimulations were performed for each MVC: the first at rest before (maximal response) and second during the plateau of MVC (superimposed response) (22). In particular, this latter was performed because the evoked response at rest may not account for changes Ia afferent pathways during voluntary contraction after stretching (10). Hence, assessing H-reflexes during MVC would be more appropriate, considering the state-dependent changes affecting Ia afferent reflex excitability and H-reflexes, such as decreases in homosynaptic depression (29) and both homonymous and heteronymous Ib inhibition (30), in addition to maintain motoneurons excitability. A rest period of 1 min was allowed before the beginning of the stretching protocol. Thereafter, a single MVC was performed, and two stimulations were delivered at the same intensity used at baseline to elicit separately each H-reflex and then each M-wave.

### Measurements

#### Ankle ROM

To monitor the changes in ankle joint ROM, a biaxial electrogoniometer (TSD 130A; Biopac System, Goleta, CA) was used. The electrogoniometer was positioned with one axis on the external face of the fibula and the other on the calcaneum. The electrogoniometer signal was transmitted to an A/D converter (mod. UM 150, Biopac System), sampled at 1000 Hz, and stored on a personal computer. The subject was prone on a medical bed. The starting ankle angle was 90°. To assess the changes in ROM, an operator slowly dorsiflexed the ankle joint manually using a visual feedback that provided her/him with angular displacement by time. The sEMG signal was checked to monitor possible muscle activation during elongation in both SM and tibialis anterior. Three trials were performed. The maximum angle reached in each set was measured to calculate the maximum ROM. The device showed very high reliability and sensitivity as reported previously (8).

#### MVC

The MVC of the plantarflexors was measured with the participant prone on a medical bed with the ankle on the ergometer and the foot fixed with a Velcro strap to a mobile metal plate instrumented with a load cell (SM-2000 N; Interface, Scottsdale, AZ). The hips and the shoulders were firmly secured to the ergometer. The participants were instructed to push as fast and hard as possible for 4 s and to focus on plantarflexion, avoiding any unnecessary movement (e.g., knee flexion and hip extension/flexion) (22). In case of any unnecessary movement, the trial was discarded, and a further attempt was performed after a 5-min rest. Visual feedback was provided to the participants. The force signal was transmitted to an A/D converter (mod. UM 150, Biopac System), sampled at a fixed sampling rate of 1000 Hz, and stored on a personal computer. The maximum force recorded was defined as the MVC and entered in the data analysis. The device showed very high reliability and sensitivity as reported previously (8).

#### sEMG recordings

sEMG signal was recorded from the gastrocnemius medialis, the gastrocnemius lateralis, and the soleus muscles. After shaving and cleaning the skin with alcohol to ensure low impedance (<5 kΩ), sEMG signals were obtained with solid hydrogel rounded electrodes (mod. H124SG Kendall ARBO; diameter 10 mm; interelectrode distance 20 mm; Kendall, Donau, Germany). The electrodes were positioned 2 cm below the insertions of the gastrocnemii over the Achille’s tendon for the *soleus*, and over the mid belly of the gastrocnemii muscles for the two gastrocnemii (31). To check for possible coactivation during passive stretching and nerve stimulation, the sEMG signal was also recorded from the *tibialis anterior* muscle (10), with the electrode placed at one-third of the distance on a line between the fibula and the tip of the medial malleolus (31). After the visual inspection of the sEMG signal, the electrodes were eventually replaced should a crosstalk contamination of the sEMG of tibial anterior occur. The sEMG signal was detected during MVC and nerve stimulation and acquired by a multichannel amplifier at a sampling rate of 2000 Hz (mod. DA100 UM 150, Biopac; input impedance, >90 MΩ; CMRR, >96 dB), amplified (gain, ×1000) and filtered (filter type IV order Butterworth filter; bandwidth, 10–500 Hz) for further analysis.

#### Nerve stimulation

**FIGURE 1:**
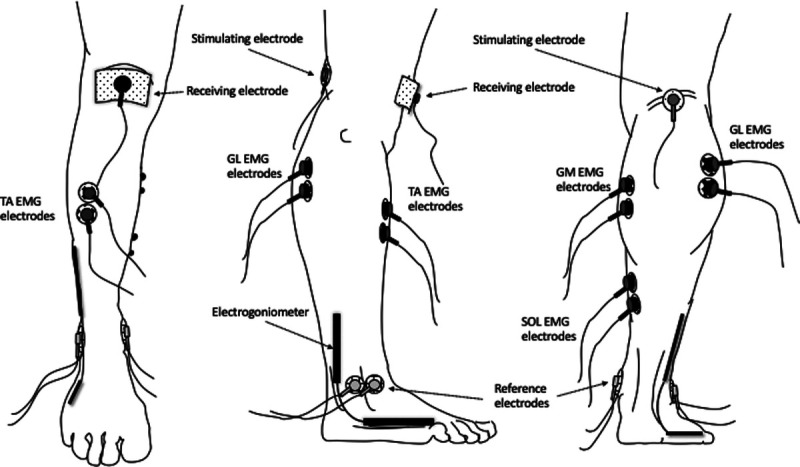
The electrode placement is shown.

Single rectangular pulses (100–150 V pulse of 1 ms in width) were delivered to the tibial nerve to evoke H-reflexes and M-waves of the plantarflexors. An adhesive cathode (8-mm diameter, Ag-AgCl) was placed in the popliteal fossa, and an anode (5 × 10 cm; Medicompex SA, Ecublens, Switzerland) was placed over the patella (Fig. 1). The electrodes were connected to a high-voltage constant-current stimulator (Model DS7AH; Digitimer Stimulator, Hertfordshire, UK). The nerve stimulation and the sEMG signal were recorded with a multichannel amplifier at a sampling rate of 2000 Hz (Mod. UM 150, Biopac System).

The stimulation intensity started from 5 mA and was progressively increased by 2 mA increments to detect the maximal H-reflex (H_max_). Thereafter, the stimulation intensity was increased by 5 mA increments to detect the maximal M-wave (M_max_) until M_max_ no longer increased. This last stimulation intensity was then increased by 20% to ensure supramaximal stimulation and used to record the maximal M-wave (M_max_). At baseline, four stimulations were performed at each intensity to build the whole recruitment curve to determine the optimal stimulation for H_max_ and M_max_ in both the control and the stretching sessions (22) before starting the passive stretching protocol. More in detail, the procedure to build such a recruitment curve was an interstimulus interval of 10s; the start of the curve was the lowest current, which was sufficient to evoke an H-reflex. The same within-subject stimulation intensities were then used to evoke the superimposed (H_sup_ and M_sup_) response, for both the baseline and the poststretching assessment (22). Because nerve stimulation was optimized for the soleus muscle (32), the H-reflex and the M-wave elicited in the gastrocnemius medialis and the gastrocnemius lateralis muscle were found not to be maximal for each participant: the H-reflex and the M-wave recorded in both gastrocnemii were found to be maximal in 24/26 participants (≈92%) and in the ascending part of the recruitment curve in the remaining 2/26 (≈8%). The same stimulation intensity was then used to evoke the superimposed responses, for both the baseline and the poststretching assessment.

A dedicated software (Mod. Stim 100C, Biopac System) enabled us to have the same stimulation order (first H-reflex, then M-wave) at a fixed temporal distance (500 ms) for each stimulation. Responses at rest were assessed without prior muscle activity at an interval of 10 s. Superimposed responses were manually triggered after the MVC plateau was attained. The V-wave was recorded during the superimposed M-wave stimulation, at the same time delay between the stimulation onset and the occurrence of the H-reflex (33).

### Data Analysis

The researchers who analyzed the data were blinded to the testing sessions (control or stretching). During the MVC, the sEMG signal was analyzed in the time domain within a 1-s period of the MVC plateau without any stimulation, and it was assessed by visual inspection. The sEMG RMS was calculated in consecutive 250-ms time windows, averaged, and then entered in the data analysis. M_max_ and M_sup_ were entered into the data analysis. The average peak-to-peak amplitudes of the evoked responses were calculated and normalized to the maximal M-wave evoked in the same condition. Thus, H_max_/M_max_, H_sup_/M_sup_, and V/M_sup_ were defined as dependent variables and were compared pre- and poststretching. The normalization of the H-reflex to the M-wave was done to provide information about the proportion of the motoneuron pool being activated in the H-reflex (34). As V-wave involves both spinal and supraspinal mechanisms, the comparison of the changes in H_sup_/M_sup_ and V/M_sup_ was proposed as a tool to estimate the relative contribution of both levels to V-wave changes (32). The M-wave accompanying the maximal H-reflex was measured and normalized to the corresponding maximal M-wave (MatH_max_/M_max_ and MatH_sup_/M_sup_) (22) to ensure the consistency of the stimulus condition during the whole experiment (35).

### Stretching Protocol

During the passive stretching protocol, the participants remained prone on the same medical bed and with the same ergometer used for the testing procedures. An operator dorsiflexed the ankle of the stretched limb until 90% of maximal discomfort, according to the subjective response for each participant. In particular, a 0–10 visual analog scale was used at this purpose, spanning from no-discomfort to maximal discomfort (5). The stretching intensity was kept constant by means of a constant force output exerted by the operator. The force output between the passively stretched leg and the operator’s arms was recorded during the protocol by a load cell (SM-2000 N; Interface, Crowthorne, UK) (5). Specifically, the load cell was positioned 5 cm above the metatarsus of the passively stretched limb, and an operator pushed perpendicularly the load cell to stretch plantarflexors. To minimize possible muscle reflex activity, muscle elongation was reached in 6 s and maintained for 45 s (28). In line with previous investigations, five 45-s sets with 15-s intervals of passive recovery were performed for a total stretching duration of 225 s (5,7,19). The sEMG signal was checked during passive stretching to monitor possible muscle activation during elongation. If the sEMG signal during the passive stretching protocol was >5% MVC, the participant was excluded from the study and replaced with another one to ensure statistical power (19). In the current study, no participant was replaced. In the control session, the participants lay prone as relaxed as possible with the ankle at a neutral angle (90°) for an equivalent duration.

### Statistical Analysis

Statistical analysis was performed using a statistical software package (IBM SPSS Statistics 22, Armonk, NY). To check the normal distribution of the sampling, the Shapiro–Wilk’s test was applied. To determine the interday reliability of the dependent parameters, the intraclass correlation coefficient and the standard error of measurement (SEM%) were calculated using the baseline values recorded during the second and third session. The intraclass correlation coefficient was interpreted as follows: very high, ≥0.90; high, 0.89–0.70; and moderate, 0.69–0.50. The minimal detectable change with a 95% confidence interval (CI) was used to detect the sensitivity of the intervention. The pre- and postdifferences in ROM and MVC in the SM and CM versus control were calculated by a three-way limb (two levels: SM and CM)–session (two levels: stretching and control)–time (two levels: pre and post) repeated-measures ANOVA. The pre- and postdifferences in sEMG RMS, sEMG RMS/M_sup_, H-reflex, M-wave, V-wave, H/M ratios, V/M_sup_, and MatH/M ratios in the gastrocnemius medialis, gastrocnemius lateralis, and soleus muscle versus the control were calculated by four-way (muscle–limb–session–time) repeated-measures ANOVA. To calculate the differences in the between-limb stretch-induced changes (SM vs CM, SM vs SM_CON_, and CM vs CM_CON_), ANCOVA was performed, assuming the baseline values as covariate. Partial eta squared (*η*_p_^2^) was calculated and classified as follows: small, <0.06; medium, 0.06–0.14; and large, >0.14 (36). Multiple comparisons were performed using Bonferroni’s correction. Significance was set at *α* < 0.05. Unless otherwise stated, descriptive statistics are presented as the mean ± SD). The changes are reported as percentage change with 95% CI. Cohen’s d ES was calculated and interpreted as follows: trivial, 0.00–0.19; small, 0.20–0.59; moderate, 0.60–1.19; large, 1.20–1.99; very large, ≥2.00 (37). The 95% CI of the ES is also reported.

## RESULTS

Table 1 presents the reliability of the dependent parameters: high to very high reliability was observed for all parameters. No change in MatH_max_/M_max_ and MatH_sup_/M_sup_ in any of the groups was noted (Table 2). Homogeneity assumption was met for all parameters.

**TABLE 1:** Interday reliability and sensitivity in the stretched and the contralateral non-SM.

		SM							Contralateral Muscle						
	Variable (mV)	Stretch	SD	Con	SD	ICC	SEM%	MDC_95%_	Stretch	SD	Con	SD	ICC	SEM%	MDC_95%_
Gastrocnemius medialis	H_max_	0.721	0.230	0.721	0.230	0.874	11.3	22.2	0.710	0.222	0.711	0.222	0.895	10.1	19.8
	H_sup_	0.704	0.229	0.723	0.235	0.910	9.8	19.1	0.710	0.223	0.713	0.227	0.904	9.8	19.2
	M_max_	4.114	1.578	4.082	1.525	0.891	12.5	24.5	4.182	1.867	4.207	1.879	0.913	13.2	25.8
	M_sup_	4.080	1.528	4.092	1.527	0.896	12.1	23.6	4.100	1.527	4.156	1.687	0.895	12.6	24.7
	V-wave	0.581	0.241	0.625	0.255	0.926	11.2	21.9	0.580	0.234	0.585	0.241	0.876	3.4	6.6
	MatH_max_	1.180	0.454	1.182	0.455	0.911	11.5	22.5	1.186	0.455	1.192	0.453	0.900	15.0	29.5
	MatH_sup_	1.190	0.467	1.187	0.460	0.888	13.1	25.6	1.197	0.476	1.205	0.469	0.896	12.6	24.6
Gastrocnemius lateralis	H_max_	0.706	0.226	0.708	0.222	0.895	10.3	20.2	0.703	0.223	0.703	0.224	0.887	10.7	21.0
	H_sup_	0.708	0.228	0.707	0.222	0.910	9.6	18.7	0.704	0.228	0.707	0.222	0.895	10.3	20.2
	M_max_	3.264	1.215	3.228	1.123	0.904	11.2	21.9	3.264	1.202	3.293	1.222	0.911	11.0	21.6
	M_sup_	3.279	1.229	3.254	1.228	0.911	11.2	22.0	3.264	1.229	3.294	1.142	0.881	12.5	24.5
	V-wave	0.533	0.214	0.498	0.221	0.934	10.8	21.3	0.534	0.202	0.537	0.209	0.899	6.2	12.1
	MatH_max_	0.950	0.363	0.932	0.335	0.895	12.0	23.6	0.963	0.368	0.970	0.399	0.903	20.4	39.9
	MatH_sup_	1.095	0.368	1.084	0.356	0.902	10.4	20.4	1.101	0.376	1.108	0.387	0.918	14.6	28.5
Soleus	H_max_	0.718	0.186	0.719	0.185	0.889	8.6	16.8	0.718	0.185	0.710	0.185	0.893	8.5	16.6
	H_sup_	0.716	0.184	0.718	0.186	0.921	7.3	14.2	0.716	0.182	0.718	0.186	0.905	7.9	15.5
	M_max_	2.164	0.699	2.162	0.696	0.915	9.4	18.4	2.159	0.675	2.170	0.715	0.929	8.6	16.8
	M_sup_	2.192	0.691	2.204	0.700	0.904	9.8	19.2	2.205	0.699	2.236	0.688	0.913	9.2	18.1
	V-wave	0.351	0.141	0.347	0.136	0.909	12.0	23.4	0.356	0.141	0.359	0.149	0.904	12.6	24.7
	MatH_max_	0.622	0.213	0.626	0.212	0.909	10.3	20.1	0.625	0.205	0.632	0.222	0.875	12.0	24
	MatH_sup_	0.644	0.215	0.639	0.211	0.913	9.8	19.2	0.645	0.209	0.649	0.218	0.898	10.5	21

H_max_, H-reflex at rest; H_sup_, H-reflex superimposed; M_max_, M-wave at rest; M_sup_, M-wave superimposed; MatH_max_, M-wave at H-reflex intensity at rest; MatH_sup_, M-wave at H-reflex intensity superimposed; ICC, intraclass correlation coefficient; SEM%, standard error of measurement as percentage; MDC_95%,_ minimum detectable change as percentage.

**TABLE 2:** Maximum (max) and superimposed (sup) H-reflex and M-wave and M-wave at H-reflex intensity over M-wave ratios (MatH/M) in the gastrocnemius medialis (GM), the lateralis (GL), and the soleus (sol) of the stretched (SM) and the contralateral (CM) limb before (pre) and after (post) a passive stretching bout or an equivalent period of rest (SM_CON_ and CM_CON_).

		SM			CM			SM_CON_			CM_CON_		
		Pre	Post	*P*	Pre	Post	*P*	Pre	Post	*P*	Pre	Post	*P*
H_max_ (mV)	GM	0.72 (0.23)	0.72 (0.24)	0.147	0.71 (0.22)	0.70 (0.23)	0.621	0.72 (0.23)	0.73 (0.24)	0.502	0.71 (0.22)	0.71 (0.21)	0.833
	GL	0.71 (0.23)	0.70 (0.22)	0.217	0.70 (0.22)	0.70 (0.23)	0.348	0.71 (0.22)	0.71 (0.22)	0.701	0.70 (0.22)	0.71 (0.23)	0.251
	Sol	0.72 (0.19)	0.73 (0.19)	0.288	0.72 (0.18)	0.72 (0.19)	0.372	0.72 (0.18)	0.72 (0.18)	0.216	0.72 (0.18)	0.72 (0.19)	0.311
H_sup_ (mV)	GM	0.70 (0.23)	0.71 (0.23)	0.373	0.71 (0.22)	0.71 (0.23)	0.930	0.71 (0.23)	0.72 (0.24)	0.269	0.71 (0.22)	0.71 (0.22)	0.850
	GL	0.71 (0.23)	0.70 (0.22)	0.110	0.70 (0.23)	0.70 (0.22)	0.898	0.71 (0.22)	0.71 (0.21)	0.950	0.70 (0.22)	0.71 (0.23)	0.415
	Sol	0.72 (0.18)	0.72 (0.19)	0.834	0.72 (0.18)	0.72 (0.18)	0.728	0.72 (0.19)	0.72 (0.18)	0.486	0.72 (0.18)	0.72 (0.19)	0.591
M_max_ (mV)	GM	4.03 (1.49)	4.07 (1.47)	0.431	4.02 (1.51)	4.06 (1.55)	0.258	4.01 (1.47)	4.03 (1.49)	0.523	4.05 (1.56)	4.04 (1.58)	0.571
	GL	3.96 (1.22)	3.95 (1.21)	0.215	3.96 (1.20)	3.97 (1.23)	0.703	3.96 (1.12)	3.94 (1.16)	0.602	3.90 (1.22)	3.91 (1.22)	0.660
	Sol	3.56 (1.10)	3.56 (1.11)	0.163	3.55 (1.11)	3.56 (1.11)	0.759	3.56 (1.10)	3.56 (1.11)	0.690	3.54 (1.11)	3.58 (1.10)	0.632
M_sup_ (mV)	GM	4.08 (1.53)	4.13 (1.50)	0.496	4.10 (1.63)	4.18 (1.64)	0.219	4.09 (1.53)	4.12 (1.51)	0.611	4.16 (1.69)	4.20 (1.76)	0.244
	GL	3.98 (1.23)	3.94 (1.20)	0.155	3.96 (1.23)	3.93 (1.27)	0.163	3.95 (1.23)	3.93 (1.15)	0.467	3.99 (1.14)	3.93 (1.22)	0.228
	Sol	3.58 (1.09)	3.60 (1.06)	0.402	3.56 (1.10)	3.55 (1.11)	0.173	3.56 (1.10)	3.52 (1.08)	0.905	3.57 (1.09)	3.56 (1.07)	0.193
MatH_max_/M_max_	GM	0.09 (0.02)	0.10 (0.02)	0.524	0.09 (0.03)	0.08 (0.01)	0.232	0.10 (0.02)	0.09 (0.01)	0.274	0.10 (0.02)	0.10 (0.02)	0.732
	GL	0.11 (0.01)	0.10 (0.04)	0.863	0.11 (0.02)	0.11 (0.02)	0.412	0.09 (0.02)	0.10 (0.02)	0.618	0.10 (0.02)	0.09 (0.01)	0.406
	Sol	0.10 (0.01)	0.11 (0.01)	0.286	0.11 (0.01)	0.11 (0.01)	0.445	0.10 (0.02)	0.11 (0.01)	0.528	0.11 (0.01)	0.10 (0.01)	0.486
MatH_sup_/M_sup_	GM	0.10 (0.03)	0.10 (0.04)	0.934	0.10 (0.04)	0.10 (0.05)	0.496	0.11 (0.03)	0.09 (0.03)	0.532	0.10 (0.04)	0.10 (0.04)	0.789
	GL	0.11 (0.03)	0.11 (0.02)	0.957	0.11 (0.02)	0.11 (0.03)	0.924	0.11 (0.02)	0.11 (0.03)	0.492	0.11 (0.01)	0.11 (0.02)	0.866
	Sol	0.11 (0.01)	0.11 (0.01)	0.715	0.11 (0.01)	0.10 (0.01)	0.830	0.10 (0.01)	0.11 (0.01)	0.285	0.10 (0.01)	0.10 (0.01)	0.976

**FIGURE 2:**
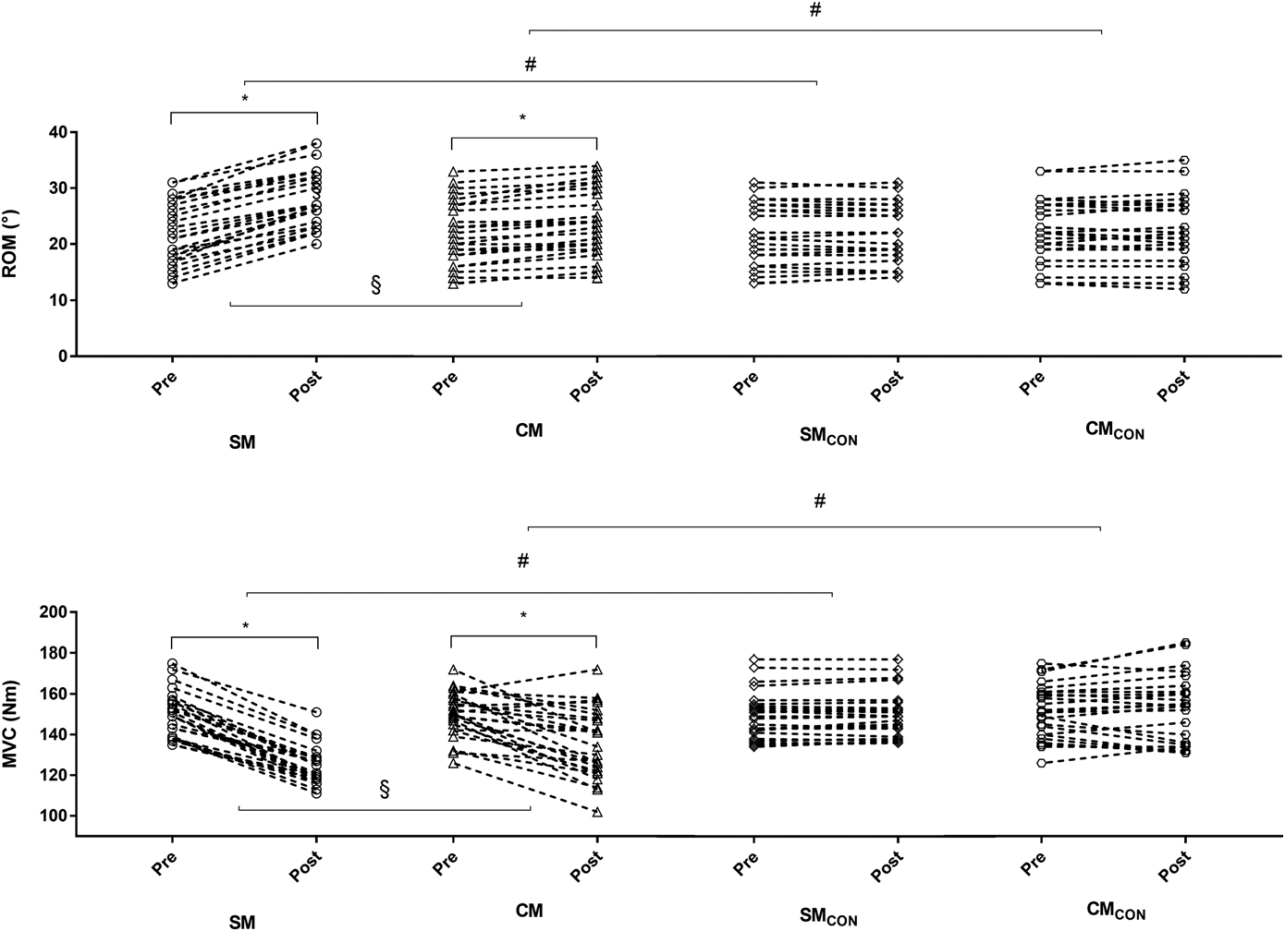
Individual data of the ankle ROM and MVC of the plantarflexor in the stretched (SM) and the CM and in the control condition (SM_CON_ and CM_CON_). *P < 0.05 post versus pre. #*P* < 0.05 versus control. §*P* < 0.05 SM versus CM.

Figure 2 presents the changes in ROM and MVC. A significant limb–session–time interaction was found for ROM (*F*_1,25_ = 14.737, *P* < 0.001, *η*_p_^2^ = 0.391). ROM was increased in the CM (8% [1%/15%], ES = 0.43 [0.02/0.84], *P* < 0.001) and SM (31% [15%/46%], ES = 1.18 [0.59/1.77], *P* < 0.001), but not in the CM_CON_ (*P* = 1.000) and SM_CON_ (*P* = 0.598). The ANCOVA showed that the increase in ROM was greater in the SM than CM (ES = 0.78 [0.20/1.33], *P* < 0.001).

A significant limb–session–time interaction was found for MVC (*F*_1,25_ = 12.777, *P* < 0.001, *η*_p_^2^ = 0.338). MVC was decreased in the CM (−9% [−21%/−2%], ES = −0.96 [−1.53/−0.38], *P* < 0.001) and SM (−17% [−22%/−12%], ES = −2.39 [−3.10/−1.68], *P* < 0.001], but not in the CM_CON_ (*P* = 0.603) and SM_CON_ (*P* = 0.192). The ANCOVA showed that the decrease in the MVC was greater in the SM than CM (ES = 0.71 [0.15/1.27], *P* < 0.001).

**FIGURE 3:**
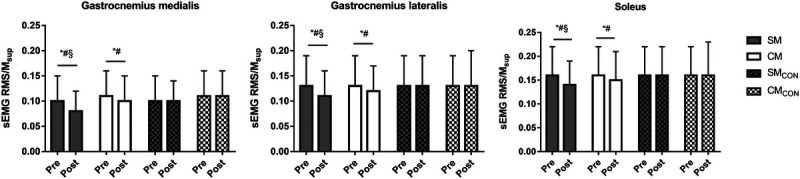
Mean(SD) of the sEMG signal RMS normalized to the superimposed M-wave (sEMG RMS/M_sup_) in the two gastrocnemii and the soleus of the stretched (SM) and the CM and in the control condition (SM_CON_ and CM_CON_). *P < 0.05 post versus pre. #*P* < 0.05 versus control. §*P* < 0.05 versus CM.

Figure 3 presents the changes in sEMG RMS/M_sup_. No muscle–limb–session–time interaction was found for the sEMG RMS/M_sup_ (*F*_2,24_ = 1.618, *P* = 0.218, *η*_p_^2^ = 0.119), although significant limb–session–time interaction was observed (*F*_1,25_ = 9.015, *P* = 0.006, *η*_p_^2^ = 0.349), indicating similar trend in all muscles. Taking all muscles together, the sEMG RMS/M_sup_ was decreased in the CM by ≈−9% (ES = −0.33, *P* < 0.05) and in SM by −17% (ES = −0.52, *P* < 0.05). No change was observed during the control session in the CM_CON_ and SM_CON_ (*P* > 0.05). The ANCOVA showed that the decrease in the sEMG RMS/M_sup_ was greater in the SM than CM (ES = 0.80, *P* < 0.05).

**FIGURE 4:**
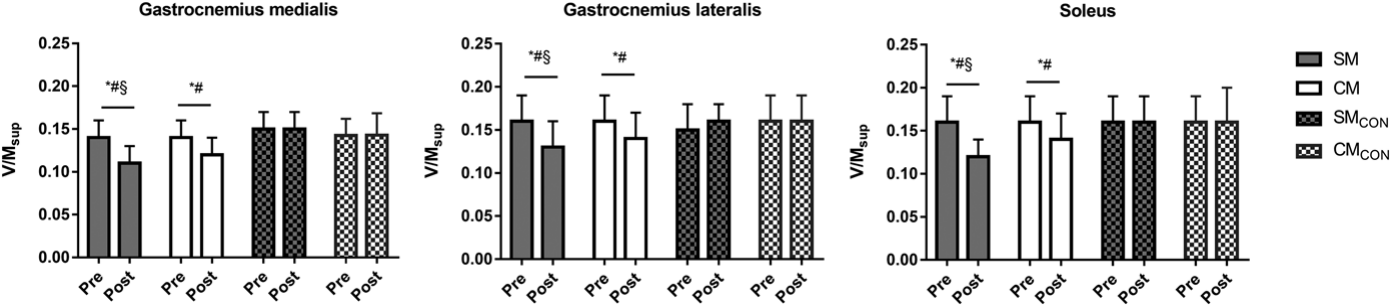
Mean ± SD of the V-wave normalized to the superimposed M-wave (V/M_sup_) in the two gastrocnemii and the soleus of the stretched (SM) and the CM) and in the control condition (SM_CON_ and CM_CON_). *P < 0.05 post versus pre. #*P* < 0.05 versus control. §*P* < 0.05 versus CM.

Figure 4 shows the reduction in V/M_sup_ in the three muscles. No muscle–limb–session–time was found for V/M_sup_ (*F*_2,24_ = 0.774, *P* = 0.472, *η*_p_^2^ = 0.021), although significant limb–session–time interaction was observed (*F*_1,25_ = 12.463, *P* = 0.002, *η*_p_^2^ = 0.333), indicating similar trends for all muscles. Taking all muscles together, the V/M_sup_ was decreased by −13% (ES = −0.81*, P* < 0.05) in the CM, whereas in the SM it was decreased by −23% (ES = −1.35, *P* < 0.05). No change was observed during the control session in the CM_CON_ and SM_CON_ (*P* > 0.05). The ANCOVA showed that the decrease in the V/M_sup_ was greater in the SM compared with CM (ES = 0.65, *P* < 0.05).

The ANOVA showed no muscle–limb–session–time for H_max_ (*F*_2,24_ = 0.553, *P* = 0.582, *η*_p_^2^ = 0.016), H_sup_ (*F*_2,24_ = 0.717, *P* = 0.498, *η*_p_^2^ = 0.019), M_max_ (*F*_2,24_ = 1.181, *P* = 0.324, *η*_p_^2^ = 0.094), and M_sup_ (*F*_2,24_ = 1.404, *P* = 0.265, *η*_p_^2^ = 0.106). Moreover, there was no muscle–limb–time interaction in H_max_ (*F*_1,25_ = 1.051, *P* = 0.365, *η*_p_^2^ = 0.078), H_sup_ (*F*_1,25_ = 0.823, *P* = 0.451*, η*_p_^2^ = 0.028), M_max_ (*F*_1,25_ = 0.847, *P* = 0.441*, η*_p_^2^ = 0.035), and M_sup_ (*F*_1,25_ = 0.467, *P* = 0.632*, η*_p_^2^ = 0.013). No change was noted in the H-reflex or M-wave in any condition in any muscle (*P* > 0.05) (Table 2). Consequently, no change in H/M ratios was observed in any condition (*P* > 0.05).

## DISCUSSION

Although the effects of passive stretching have been extensively investigated in the SM, the current study sought to examine the effects of passive stretching on the neuromuscular mechanisms influencing the force-generating capacity of the CM. After a passive stretching protocol, the ROM was increased, and the MVC, the sEMG RMS, and the V/M_sup_ were decreased in the CM, whereas no change in the H/M ratios or the M-wave was observed. The decrease in V/M_sup_ and the concomitant lack of change in H/M ratios and M-wave suggest that supraspinal rather than spinal and peripheral mechanisms could underlie the drop in the force-generating capacity of the CM.

### Preliminary considerations

No change in any parameter was observed in the SM_CON_ and CM_CON_. In addition, the high to very high reliability recorded here indicates that the observed findings are attributable mainly to the intervention and not to the low reproducibility of the protocols. The MatH accompanying the H-reflex ensured consistency in nerve stimulation (35). The similar pre- and post-MatH/M ratios indicated that the H-reflex was recorded within the same portion of the recruitment curve, with stable nerve stimulation (22).

To verify the effectiveness of the stretching protocol, we assessed the ROM, MVC, sEMG RMS, and nerve stimulation in the SM as well. In line with previous studies, the ankle ROM was increased, whereas the MVC and the sEMG RMS of the plantarflexors were decreased in the SM (8,19,28). The decrease in the sEMG RMS/M_sup_ observed here (38,39), together with the decrease in the V/M_sup_ without any simultaneous change in H/M ratios and M-wave, may indicate that the supraspinal activity was affected, in line with what was previously shown (23,38). Although, the supraspinal level cannot be clearly identified with the present design. For instance, recent studies using transcranial magnetic stimulation to investigate modifications in cortical activity after passive stretching found no change (10,40–42). Therefore, excluding the changes at the cortical level, it is likely that the efferent drive generated at the subcortical level (i.e., basal ganglia, cerebellum, and ventral–anterior and ventral–lateral thalamus nuclei) could have been affected by passive stretching (43). The lack of change in the H/M ratios indicates that passive stretching does not impair the spinal contribution to the force-generating capacity (9). Similarly, a recent study observed no change at the spinal level after passive stretching (10). Although the role of the muscle spindles and of the free nerve endings was hypothesized to affect the force-generating capacity at the spinal level (9), it is possible that the present procedures were not adequate to capture these changes, as recently acknowledged (10). Indeed, the H-reflex bypasses the muscle spindles, so that it is not influenced by their sensitivity (10). Nonetheless, it could still be hypothesized that stretching might reduce the intrafusal muscle spindle discharge through a muscle spindle desensitization, albeit without any change in the H-reflex (3,10,41). In line with the literature, no change in the M-wave was observed, indicating that the decrease in the force-generating capacity cannot be ascribed to any peripheral mechanism (e.g., impaired propagation of the neuromuscular action potential or excitation–contraction coupling) (9,10,19,44).

### Stretch-induced changes in the CM

The observation of a small increase in ROM in the CM is shared by previous reports (11–14,19). An increase in ROM is attributable to a decrease in muscle–tendon unit stiffness, which depends on the following: (i) the cross-link between actin and myosin filaments (45); (ii) the noncontractile proteins of the endosarcomeric (46) and the exosarcomeric cytoskeleton (47); (iii) the changes in the viscoelastic properties of the connective tissue located within and surrounding the muscle (46); and (iv) an augmented stretch tolerance during passive stretching, involving a reduction in muscle tone possibly associated with a reduction in nociceptive activity (48). The first three factors hark back to mechanical stimuli, in which the CM was not involved. Consequently, the augmented stretch tolerance is the only factor that could be hypothesized because it is the only factor involving neuromuscular pathways (19).

The passive stretching protocol led to a moderate decrease in MVC in the CM. Inconsistent results for this phenomenon have been reported because some studies showed a decline in maximal force (17–19), whereas others did not (13,14,16). Such a discrepancy may derive from the muscle stretched, the stretching modality (e.g., passive or dynamic stretching), the overall duration of the passive stretching protocol, and the stretching intensity (9). In particular, it should be acknowledged that the duration of the present stretching protocol might be longer than what actually happen in the practice (49,50) so that the effects might be greater. The force-generating capacity depends on both neuromuscular and mechanical factors. A recent study showed a decrease in sEMG RMS as a gross neuromuscular process and excluded the mechanical factors as possible mechanisms in the CM (19). Because the sEMG is the gross sum of the central and peripheral contribution to the skeletal muscle activation, the present procedures enabled us to better detect the origin of the neuromuscular mechanisms and possibly identify whether supraspinal, spinal, and/or peripheral mechanisms were involved.

Given the normalization of all parameters to the M-wave amplitude, it is important to note that no change in the M-waves was observed in the gastrocnemius medialis, gastrocnemius lateralis, or soleus muscles, whereas the sEMG/M_sup_ was decreased in all muscles. This means that the peripheral neuromuscular mechanisms, associated with the propagation properties of the nerve and sarcolemmal action potential and with the excitation–contraction coupling, were not affected by the passive stretching protocol (19). As such, the changes in the sEMG/M_sup_ may be ascribed to central neuromuscular factors (10,38). Because the V-wave and the H-reflex were normalized to the M-waves, the possible changes in the V/M_sup_ and H/M ratios could be explained by supraspinal and/or spinal mechanisms.

As hypothesized, we observed no change in the H/M ratios in the CM. The lack of change in H/M_max_ suggests that no change in homosynaptic depression (29) or homonymous and heteronymous Ib inhibition (30) have occurred. Moreover, the unchanged H/M_sup_ points out that the an alteration in the Ia afferent pathways during the voluntary contraction after passive stretching did not occur (10). This is in line with the only previous study that assessed the spinal contribution to the CM after passive stretching and reported no change in the spinal reflex excitability (27). Such a lack of change in spinal reflex was observed after a shorter and less intense passive stretching protocol and technique (transcutaneous spinal cord stimulation) (27). Hypothetically, a change in the spinal contribution might be ascribed to a change in a motoneuron facilitatory mechanism from the neuromuscular spindles in the SM toward the motoneurons in the CM via the interneurons interposed in the crossed pathway (20,39,51,52). Such a change in motoneurons facilitatory mechanism may be mediated by two possible factors: (i) an alteration in the excitability of the Ia afferent-motoneuron reflex pathway to transmit muscle spindle activity and (ii) a decrease in the intrafusal muscle spindle discharge through desensitization of the muscle spindle, thus decreasing motoneuron activity (10,39,51). Because no mechanical stimulus and response occurred in the CM (19), the muscle spindle desensitization is not expected to be involved. However, the present results also indicate that the former mechanism was not even involved.

Moderate decreases in the V/M_sup_ in all CM were found. A decrease in V-wave amplitude without any concomitant change in H/M_sup_ would point out a reduced motoneuron recruitment or firing rate, which may indicate lower supraspinal contribution to the motoneuron pool (53). Notwithstanding, the motoneuron firing rate is an index of not only the supraspinal input to the motoneuron but also the response to all inputs to the motoneuron, thereby making a clear identification of the origin of a change in V-wave amplitude difficult to achieve (25). Because these procedures were used here for the first time in the CM, a direct comparison with the literature cannot be made. Using a different approach (i.e., interpolated-twitch technique), voluntary activation was reduced after passive stretching in the CM, indicating a reduction in the neural central drive (19). However, voluntary activation does not permit distinguishing the supraspinal from the spinal neuromuscular mechanisms (21). In addition, neither the V-wave nor the voluntary activation can indicate accurately whether the changes occur at the cortical and/or the subcortical level (54). Nevertheless, previous studies reported no change in motor-evoked potential in the motor cortex activating the SM, indicating that passive stretching does not impair the cortical activity (40,41), making it unlikely that a change in the motor cortex activates the CM. Excluding any change at the cortical level, it is likely that subcortical mechanisms might be responsible for the drop in the V-wave. The subcortical neural patterns associated with the cortical–subcortical circuits between the basal ganglia and the cerebellum have been suggested as possible pathways that allow the crossover of the afferent stimuli from the muscle spindles, mechanoreceptors, and metabo-/nociceptors (43). Lastly, it was shown that passive stretching could affect the monoaminergic (e.g., norepinephrine and serotonin) drive in a nonlocalized way via an increase in parasympathetic activity (55), possibly reducing the force-generating capacity of the CM (9,39). It should be noted that the changes in the CM had lesser extent than in the SM, as recently shown (19). The inhibitory interneurons within the subcortical circuits may have mediated such a lower crossover efferent response toward CM (56).

The present study has several limitations. First, the cortical mechanisms were not investigated. We acknowledge that different methods (e.g., transcranial magnetic stimulation or electrical stimulation of the cervicomedullary junction) could have deepened the supraspinal mechanisms. Future studies are needed to explore these possible mechanisms in the CM. Second, the H-reflex is a product of small proportion of motoneurons so that undetectable changes are still possible (25). Third, no information about the role played by the peripheral receptors is provided. Lastly, the current procedures were conducted at a neutral ankle angle position (i.e., 90°), and assessing the procedures at shorter or longer muscle lengths could result in different outcomes.

## CONCLUSION

The present study was conceived to investigate the effects of unilateral passive stretching on the neuromuscular mechanisms behind the reduction in the force-generating capacity of the CM. The decrease in the MVC and the sEMG RMS of the CM was accompanied by a decrease in the V/M_sup_ but not the H/M ratios and the M-wave, suggesting that only supraspinal mechanisms might be involved in the contralateral decrease in the maximum force-generating capacity.
